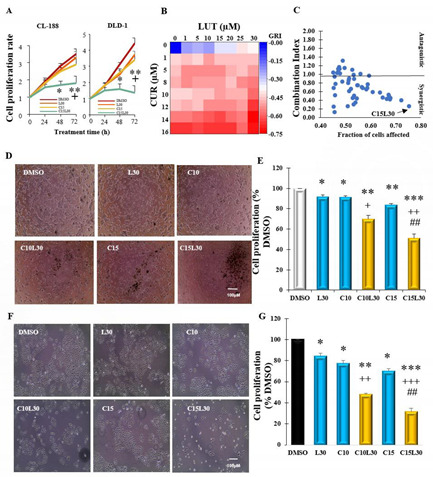# Correction: Aromokeye, R.; Si, H. Combined Curcumin and Luteolin Synergistically Inhibit Colon Cancer Associated with Notch1 and TGF-β Signaling Pathways in Cultured Cells and Xenograft Mice. *Cancers* 2022, *14*, 3001

**DOI:** 10.3390/cancers14174238

**Published:** 2022-08-31

**Authors:** Rukayat Aromokeye, Hongwei Si

**Affiliations:** Department of Human Sciences, Tennessee State University, Nashville, TN 37209, USA

## Error in Figure

In the original publication [1], there was a mistake in Figure 1A as currently published: The Figure 1A was accidentally misused from our other similar study. Although the wrong figure cannot affect the conclusion of this paper, we have to use the correct one for this publication. The corrected Figure 1 appears below. The authors apologize for any inconvenience caused and state that the scientific conclusions are unaffected. This correction was approved by the Academic Editor. The original publication has also been updated.