# Supplementary material for: Combined Curcumin and Luteolin Synergistically Inhibit Colon Cancer Associated with Notch1 and TGF-β Signaling Pathways in Cultured Cells and Xenograft Mice
Source: Cancers (Basel). 2022 Jun 18;14(12):3001. doi: 10.3390/cancers14123001 (PMC9221484; doi:10.3390/cancers14123001)
Supplement: Supplementary file 1 [file cancers-14-03001-s001.zip › cancers-1659134-supplementary.pdf]

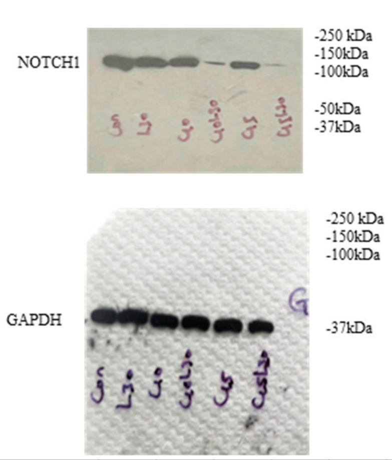

Average Intensity	DMSO	L30	C10	C10L30	C15	C15L30
NOTCH1	29537.1	22878.7	18952.1	12886.7	16370	9403.92
GAPDH	36290	34502.5	33582.7	29627.5	29711.7	28748.3
Ratio (NOTCH1/GAPDH)	0.81392	0.6631	0.56434	0.43496	0.55096	0.32711

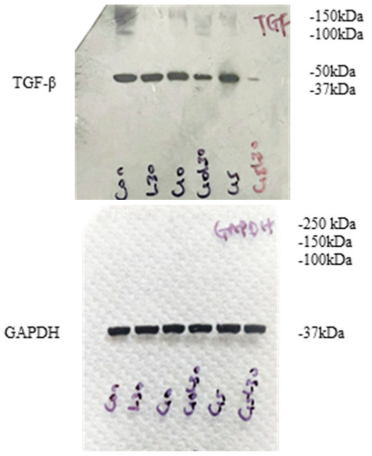

Average Intensity	DMSO	L30	C10	C10L30	C15	C15L30
TGF-β	26346	22850.8	22046.4	14631.9	17502.3	9391.91
GAPDH	26191.3	24576.9	25728.6	23990.8	22859.5	23552.1
Ratio (TGF-β/GAPDH)	1.00591	0.92977	0.85688	0.60989	0.76565	0.39877

Figure S1. Uncropped western blot images in Figure 3A,B.

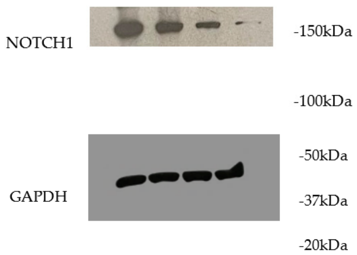

Average Intensity	DMSO	L30	C10	C10L30	C15	C15L30
NOTCH1	29537.1	22878.7	18952.1	12886.7	16370	9403.92
GAPDH	36290	34502.5	33582.7	29627.5	29711.7	28748.3
Ratio (NOTCH1/GAPDH)	0.81392	0.6631	0.56434	0.43496	0.55096	0.32711

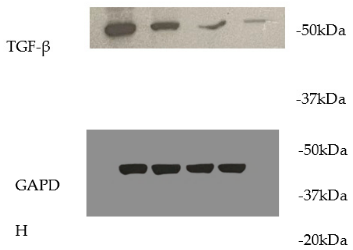

Average Intensity	DMSO	L30	C10	C10L30	C15	C15L30
NOTCH1	29537.1	22878.7	18952.1	12886.7	16370	9403.92
GAPDH	36290	34502.5	33582.7	29627.5	29711.7	28748.3
Ratio (NOTCH1/GAPDH)	0.81392	0.6631	0.56434	0.43496	0.55096	0.32711

Figure S2. Uncropped western blot images in Figure 4E,F.
